# Supplementary material for: The kinase LYK5 is a major chitin receptor in Arabidopsis and forms a chitin-induced complex with related kinase CERK1
Source: eLife. 2014 Oct 23;3:e03766. doi: 10.7554/eLife.03766 (PMC4356144; doi:10.7554/eLife.03766)
Supplement: Supplementary file 1. — Primer sequences used in this study. DOI: http://dx.doi.org/10.7554/eLife.03766.022 [file elife03766s001.pdf]

Primer	sequence (5'-3')
CERK1F	GGGGACAAGTTTGTACAAAAAAGCAGGCTccATGAAGCTAAAGATTTCTCTAAT
CERK1R	GGGGACCACTTTGTACAAGAAAGCTGGGTtCCGGCCGGACATAAGACTGACT
LYK2F	GGGGACAAGTTTGTACAAAAAAGCAGGCTCCATGGCTGTTTCAGTTAGTAAGC
LYK2R	GGGGACCACTTTGTACAAGAAAGCTGGGTtATCTATTATACTACTCTTCTTTAC
LYK3F	GGGGACAAGTTTGTACAAAAAAGCAGGCTccATGAATCTTACCTTCTACATCTTC
LYK3R	GGGGACCACTTTGTACAAGAAAGCTGGGTtTCTTCCTTGGACTAGACCACT
AtLYK4-F	GGGGACAAGTTTGTACAAAAAAGCAGGCTCCATGATCTCGTTTTCATTTC
AtLYK4-R	GGGGACCACTTTGTACAAGAAAGCTGGGTTGTACGACGATTCTTCCCAG
LYK5F	GGGGACAAGTTTGTACAAAAAAGCAGGCTCCATGGCTGCGTGTACACTCCACG
LYK5R	GGGGACCACTTTGTACAAGAAAGCTGGGTtGTTGCCAAGAGAGCCGGAACGAAG
LYK5-KinF	TCGGAATTCTCATTACCGTCGTCGTTGCC
LYK5-KinR	GGTGCTCGAGGTTGCCAAGAGAGCCGGAACG
LYK5-K395EF	GGCGACGATGCCGCTGTGGAAGTGATCAAAGGAGATG
LYK5-K395ER	CATCTCCTTTGATCACTTCCACAGCGGCATCGTCGCC
PromlyK5-1.8KF	GGGGACAAGTTTGTACAAAAAAGCAGGCTCCCCACAGTTATCTCTGGTTTGATTTAG
LYK5-NoKin-R	GGGGACCACTTTGTACAAGAAAGCTGGGTTGAGCTTGTTCTCCTCCGGCAAC
CERK1-KDF	CTGAGAATTCTATGCTTACCGGAAGAATAAG
CERK1-KDR	ACGTGTCGACCTACCGGCCGGACATAAGACTGAC
LYK5-T72GF	CAACCACCGTACAACGGGGCGGACTCAATCG
LYK5-T72GR	CGATTGAGTCCGCCCCGTTGTACGGTGGTTG
LYK5-S206PF	CCATGGGAGATAGTATCCCCGGCATCGCCGAGATG
LYK5-S206PR	CATCTCGGCGATGCCGGGGATACTATCTCCCATGG
LYK5-S216PF	GATGTTCAACAGCACACCCGCCGCCATAACCGAAG
LYK5-S216PR	CTTCGGTTATGGCGGCGGGTGTGCTGTTGAACATC
LYK5-Y128GF	CCAACACAACGCCACTGGCAATCTCTCCGGTAAC
LYK5-Y128GR	GTTACCGGAGAGATTGCCAGTGGCGTTGTGTTGG
pHisThrom-F	GTTTCCAGTCTCTGGTTCCGCGTGGATCTCACCACCATCATCATCACTGG
pHisThrom-R	TGATGGTGGTGAGATCCACGCGGAACCAGAGACTGGAAACGCACGGTTTC
LYK5EctoF	TACTTCCAATCCAATGCCCAGCAACCGTACGTCAACAACCA
LYK5EctoR	TTATCCACTTCCAATGTTATTTGTGAGAAGAAGAAGATCCCGGAG
CERK1EctoF	CGCGGATCCAAGTGCAGGACTAGCTGTCCTTTA
CERK1EctoR	CGCGTCGACTTAACCAACACCATCTTGTTTACTTGA
WRKY29F	ATCCAACGGATCAAGAGCTG
WRKY29R	GCGTCCGACAACAGATTCT
WRKY30F	AGCCAAATTTCCAAGAGGAT
WRKY30R	GCAGCTTGAGAGCAAGAATG
UBQ10F	GGCCTTGTATAATCCCTGATGAATAAG
UBQ10R	AAAGAGATAACAGGAACGGAAACATAGT
WRKY29F	ATCCAACGGATCAAGAGCTG
WRKY29R	GCGTCCGACAACAGATTCT

WRKY30F	AGCCAAATTTCCAAGAGGAT
WRKY30R	GCAGCTTGAGAGCAAGAATG
UBQ10F	GGCCTTGTATAATCCCTGATGAATAAG
UBQ10R	AAAGAGATAACAGGAACGGAAACATAGT
WRKY33F	GGTCACAACAATCCGGAAGA
WRKY33R	GGAGAGACAAGAGAAGGAGAGA
WRKY53F	TCACCGAGCGTACAACTTATTCC
WRKY53R	CGTTTATCGATGCCGGAGATT
LYK5-qRT-F	ATCGTACCTCGTCTTCGAGTAT
LYK5-qRT-R	TGCCACATCCCTTGCTATTT
LYK4-qRT-F	GGTTGAGACGAGCAAGACTTTA
LYK4-qRT-R	GAGGATTCACCAAAGGGATCAG
CERK1-qRT-F	AAGACAAAGATAGAATCCAGACGGGCTCTAGGG
CERK1-qRT-R	TCCGCGACTCTTTCGTAAGTATCTTCCTGT

Table supplement 1. Primers used in this study.
